# Relationship between maternal pelvis height and other anthropometric measurements in a multisite cohort of Ugandan mothers

**DOI:** 10.11604/pamj.2016.24.257.9889

**Published:** 2016-07-20

**Authors:** Ian Guyton Munabi, Josaphat Byamugisha, Livingstone Luboobi, Samuel Abilemech Luboga, Florence Mirembe

**Affiliations:** 1 Department of Human Anatomy, School of Biomedical Sciences, Makerere University College of Health Sciences, New Mulago Hospital Complex, Kampala Uganda; 2 Department of Obstetrics and Gynecology, School of Medicine, Makerere University College of Health Sciences, New Mulago Hospital Complex, Kampala Uganda; 3 Department of Mathematics, Makerere University College of Natural Sciences, Makerere University, Kampala, Uganda

**Keywords:** Maternal pelvis height, childbirth, anthropometry, screening

## Abstract

**Introduction:**

In sub Saharan Africa, childbirth remains a challenge that creates the need for additional screening tools. Maternal pelvis height, which is currently in use by automotive engineers has previously been shown to have significant associations with various childbirth related outcomes and events. This study set out to determine the associations between maternal: Age, height, weight and number of pregnancies with maternal pelvis height in Ugandan mothers.

**Methods:**

This was a secondary analysis of maternal birth records from nine Ugandan hospitals, of mothers with singleton pregnancies. Data was analyzed using multilevel regression with respect to maternal pelvis height and additional analysis for tribe and site of childbirth intraclass correlations (ICCs).

**Results:**

The mean maternal pelvis height was 7.30cm for the 2068 records. Maternal pelvis height was associated with: a 0.01cm reduction per centimeter of maternal height (P=0.02), 0.01cm increase per kg of maternal weight (P<0.01), 0.04cm increase for each additional pregnancy (P=0.03) and 0.03cm increase with respect to tribe of mother (P=0.27), for a constant of 7.97cm (P<0.01). The ICC for tribe was 0.20 (SE=0.08) and 0.37 (SE=0.11) for site.

**Conclusion:**

Maternal pelvis height was associated with maternal height, maternal weight and number of pregnancies. The site of childbirth had a moderate effect on the above associations with maternal pelvis height. More study on the public health screening value of these measurements in these settings is required.

## Introduction

The loss, misery and pain as a result of maternal death and the related stillbirth remain a challenge for most of sub-Saharan Africa [1]. Recent estimates suggest that 98% of the global stillbirths occur in low and middle-income countries, which includes most of sub-Saharan Africa [2]. The combination of poor health information systems, weak health systems and low facility utilization on the part of mothers only serve to further hide the true magnitude of this challenge [3].

Despite these challenges it is important to note that in the case of Uganda, 94% mothers will come to a health facility at least once during the antenatal period of their pregnancy [4]. This single contact with the health care system is thus an important screening opportunity in these settings. Sadly in most sub-Saharan African, the challenges associated with lack of access to regular power supply make it difficult or impossible to use modern tools, like ultra-sonography, for assessing risk of adverse pregnancy outcomes during the antenatal period. The absence of appropriate screening tools, coupled with populations where the girl children are still exposed to poor early childhood nutrition which in turn leads to short and or stunted women [5], means that for many mothers the above mentioned screening opportunity is lost. As result, these short and or stunted women may later develop difficulties during childbirth [5–7].

**Figure 1:**
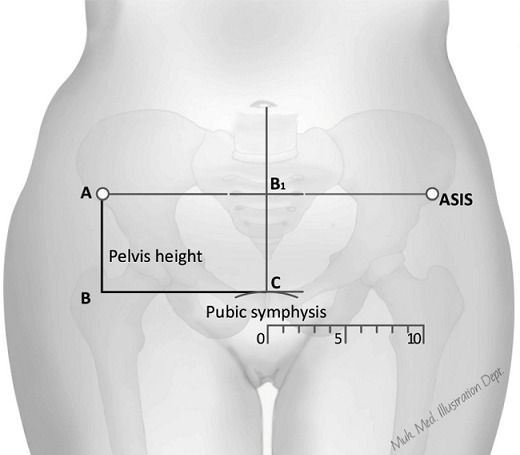
Measurement of pelvis height

In a series of earlier studies we have demonstrated that maternal pelvis height, when used in combination with maternal height and maternal weight increases the chances of catching mothers with adverse outcomes of pregnancy [8, 9]. Elsewhere we have shown that reducing maternal pelvis height is associated with an increased risk of fetal head molding [10]. We also argue that the maternal pelvis height is a better measure of a mother's birth canal dimensions in view of: (a) the unique growth pattern of the female pelvis that continues till the age of 30 and (b) its closer relationship with other birth canal dimensions [8, 11]. It is important to note that Pelvis height is currently used by automotive vehicle engineers to mark off the contribution pelvis bones to total height in crash test dummies [12]. Pelvis height was obtained using two easy to identify bony landmarks (Symphysis pubis and Anterior Superior Iliac spines) as shown in Figure 1.

The research leading to this manuscript was driven in part by the need for appropriate and generalizable additional screening tools to make the most of the screening opportunity made by the mother's single contact with the health care system during pregnancy. The research also sought to further define the observed clustering effect, which has been described by others [13], of maternal anthropometric measurements and outcomes of pregnancy around the site of childbirth [8]. This hypothesized clustering when coupled with a hypothesized low mobility of communities in rural settings creates an opportunity for use of individual anthropometric measurements, in defining public health trends related to childbirth. This study set out to determine the associations between maternal: Age, height, weight and number of pregnancies with maternal pelvis height in Ugandan mothers.

## Methods

**Figure 2:**
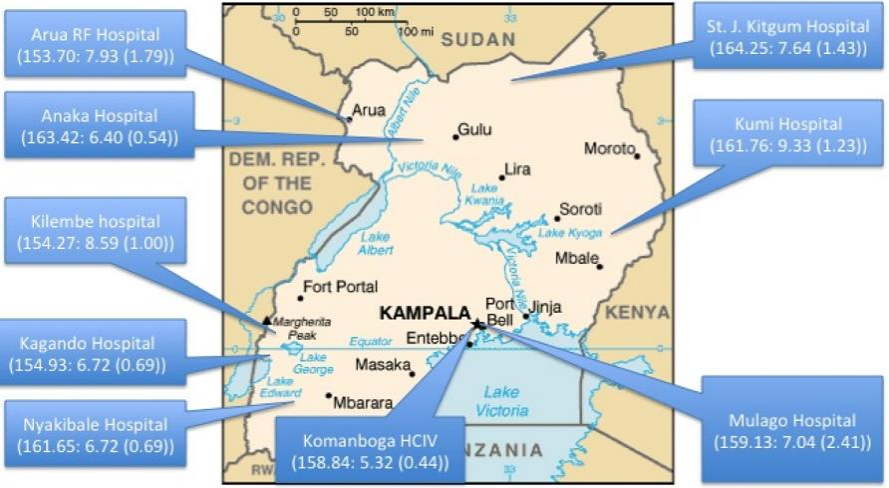
Showing study sites with corresponding (mean maternal height: mean pelvis height (SD))

This was a secondary analysis of data from questionnaires administered to 2068 mothers at various stages of pregnancy from various sites in Uganda. There were 387 (18.71%) records from Mulago hospital in central Uganda, 188 (9.09%) Komamboga hospital, 377 (18.23%) records from St. Joseph Hospital Kitgum in Northern Uganda, 86 (4.16%) from Anaka hospital in Northern Uganda, 366 (17.70%) Arua hospital in Northwestern Uganda, 352 (17.02%) from Kagando hospital in Western Uganda, 116 (5.61%) from Kilembe hospital in Western Uganda, 46 (2.22%) from Nyakibaale hospital in Southwestern Uganda and 150 (7.25%) records from Kumi Hospital in Eastern Uganda. Figure 2 is a representative map of Uganda showing the locations of the above participating sites. The selection criteria for mothers in the parent study included: carrying a single fetus, with a vertex presentation. Table 1, which provides a summary of the descriptive statistics for the study population.

**Table 1:** Descriptive statistics for the study population

Variable (Unit)	No.of records	Mean (SD)
Age (years)	2068	25.52 (5.74)
Height (Centimeters)	2012	158.56 (8.62)
Weight (Kilograms)	2029	62.27 (9.78)
Gravidity	2068	3.08 (2.14)
Pelvis height (Centimeters)	2068	7.30 (1.81)

The target sample size of 2150 records was obtained using the sample size formula for estimated sample size for one-sample comparison of mean available as part of the sampsi function of STATA 13. The sample size was calculated based on the following assumptions: alpha =95%, beta =0.05, previously observed mean for maternal pelvis height of 7.32 Cm [8], a standard deviation of 1.89 and another hypothesized mean of 7.50 Cm from literature [14]. This gave a total of 1433 records, which was inflated by a 1.5 allowance for the design effect of recruiting mothers from multiple sites to give the final target sample size of 2150 records. For each record we obtained information on: site, maternal age, maternal weight, gravidity, and maternal height. A record was also made of the maternal pelvis height by each of the participating midwives who had been trained in how to make this measurement using a pair of rigid rulers placed at right angles on the pubis Symphysis and anterior superior iliac spines as shown in Figure 1.

Descriptive statistics were generated using means with additional comparisons for differences between sites and maternal tribe using ANOVA. Inferential statistics were generated first by ordinary regression analysis followed by multilevel regression analysis using the xtmixed function in STATA 13 (StataCorp LP, Texas, USA) to control for clustering on mother tribe and the different sites. Stepwise backward multilevel regression was used to identify the significant variables of interest in the final model with respect to maternal pelvis height. Additional comparisons of model clustering, was made using the intraclass correlation (ICC) coefficient obtained using the estat icc function in STATA. Maternal height was retained in the final model as one of the key anthropometric measurements under study. During analysis records with missing values were dropped. The level of significance was set as P<0.05 for all statistical tests.

Ethical considerations for the parent study included obtaining ethical approval from the Makerere University School of Biomedical Science Institutional Review Board and the proposal for the larger study in which this one was nested was registered with the Uganda National of Science and Technology. As part of the approval process the study was registered with the president's office and a letter of introduction provided to inform the local leaders in the districts. The hospital administrators and heads of units were briefed of the parent study and the need to obtain a copy of the maternal birth records. All the participating nursing staff were verbally requested to be part of the study and offered an equivalent of 1USD compensation for each birth record completed. Each mother in the parent study was informed about the study and requested to consent to participate in the study. With the exception of measuring maternal pelvis height there were no other procedures or modifications made to the current birthing practice at any of the participating sites. No identifier marks of personal information were used in the analysis and subsequent reporting of the study results.

## Results

In Table 1 the mean value for maternal pelvis height was 7.30 Centimeters with a standard deviation of 1.81 for 2068 records. These 2068 records correspond to 96.18% of the targeted 2150 records that gave a new improved power of 0.998 for the study. Table 2, compares the averages for of the following variables: age, height, weight, gravidity, and maternal pelvis height by each of the participating study sites. In this Table 2, there were significant differences in the means of each of the study variables across the various sites. Table 3 compares the averages of the same set of variables across the different tribes. In this table there were also significant differences for all the study variables.

**Table 2:** Showing the comparison of means for the different variables by study site

Variable (Units)	Mean (Standard deviation)										
Site	Overall	Mulago	Kagando	Kilembe	Nyakibaale	Anaka	Kitgum	Arua	Kumi	Komamboga	ANOVA (P value)
Age (years)	25.52 (5.74)	25.17 (4.67)	26.05 (5.59)	25.24 (6.86)	27.63 (5.21)	25.72 (6.71)	25.64 (5.76)	24.52 (6.36)	25.80 (6.77)	26.32 (4.32)	**3.34 (<0.01)**
Height (Centimeters)	158.56 (8.62)	159.13 (8.17)	154.93 (4.90)	154.27 (5.33)	161.65 (5.82)	163.42 (6.35)	164.25 (6.11)	153.70 (11.23)	161.76 (8.58)	158.84 (6.24)	**65.05 (<0.01)**
Weight (Kilograms)	62.27 (9.78)	66.72 (9.49)	56.45 (6.61)	58.36 (10.86)	64.35 (6.98)	59.56 (7.74)	64.95 (9.07)	60.95 (8.78)	61.29 (12.07)	64.79 (10.38)	**39.58 (<0.01)**
Gravidity	3.08 (2.14)	2.27 (1.23)	3.33 (2.44)	3.08 (2.41)	3.91 (2.14)	3.44 (2.37)	3.32 (2.25)	2.98 (1.95)	3.88 (2.94)	2.97 (1.44)	**12.68 (<0.01)**
Pelvis height (Centimeters)	7.30 (1.81)	7.04(2.41)	6.72 (0.69)	8.59 (1.00)	6.33 (0.54)	6.40 (0.77)	7.64 (1.43)	7.93 (1.79)	9.33 (1.23)	5.32 (0.44)	**106.81 (<0.01)**

**Table 3:** Showing the comparison of means for the different variables by the different tribes

Variable (Units)	Mean (Standard deviation)										
Tribe	1	2	3	4	5	6	7	8	9	10	ANOVA (P value)
Age (years)	24.45 (6.38)	25.47 (4.69)	26.04 (5.14)	25.26 (4.61)	26.40 (3.70)	25.66 (5.97)	26.80 (4.64)	25.74 (6.64)	25.79 (5.77)	26.30 (5.66)	**2.10 (0.03)**
Height (Centimeters)	153.46 (11.19)	158.74 (7.73)	160.57 (6.87)	158.42 (6.78)	163.65 (6.83)	164.09 (6.18)	157.60 (6.85)	161.95 (8.31)	154.05 (4.08)	161.03 (7.63)	**65.58 (<0.01)**
Weight (Kilograms)	60.82 (8.81)	66.22 (10.36)	65.90 (8.91)	62.48 (8.44)	66.33 (6.91)	64.03 (9.13)	65.17 (8.34)	61.69 (11.60)	55.82 (6.28)	66.09 (10.42)	**37.54 (<0.01)**
Gravidity	2.97 (1.97)	2.47 (1.33)	2.80 (1.76)	2.41 (1.31)	3.07 (1.44)	3.35 (2.27)	2.57 (1.22)	3.74 (2.90)	3.26 (2.41)	3.35 (2.27)	**7.35 (<0.01)**
Pelvis height (Centimeters)	7.94 (1.77)	6.42 (2.10)	6.94 (2.04)	6.05 (1.82)	6.73 (2.57)	7.41 (1.43)	6.81 (2.11)	9.20 (1.37)	7.06 (1.07)	6.96 (1.84)	**45.43 (<0.01)**

**Key: 1-Lugbara/Alur/Kakwa, 2-Ganda, 3-Ankole, 4-Soga, 5-Gishu, 6-Acholi/Lango, 7-Nyoro/Toro, 8-Iteso, 9-Konjo, 10-others**

Significant large pair wise correlations were observed between maternal age and gravidity (0.70, P<0.01). Moderate correlations were observed between maternal height and maternal weight (0.39, P<0.01). There were small significant correlations between the other pairs of variables: maternal height and maternal age (0.15, P<0.01), maternal age and maternal weight (0.19, P<0.01), number of pregnancies (gravidity) and maternal height (0.13, P<0.01), number of pregnancies and maternal weight (0.09, P<0.01) and number of pregnancies and maternal pelvis height (0.08, P<0.01). Table 4 shows the results of both the uni-variable and multivariable regression modeling analysis of the different study variables against maternal pelvis height. On uni-variable modeling it was only number of pregnancies (gravidity) and site of childbirth that had a significant effect on maternal pelvis height (Table 4). In Table 4 all the constants on uni-variable modeling were significant with P values<0.01.

**Table 4:** Showing regression modeling of the study variables and maternal pelvis height

Variable	Uni-variable modeling: Coef. (95% CI, P value, constant)	Multivariable modeling: Coef. (95% CI, P value)+
Age	0.003 (-0.01 to 0.02, 0.62, 7.26)	-
Height	-0.002 (-0.01 to 0.01, 0.67, 7.61)	-0.01 (-0.02 to -0.002, 0.02)
Weight	0.007 (-0.001 to 0.02, 0.07, 6.84)	0.01 (0.01 to 0.02, <0.01)
Gravidity	0.07 (0.03 to 0.10, **<0.01**, 7.09)	0.04 (0.004 to 0.07, 0.03)
Site	0.04 (0.02 to 0.06, **< 0.01**, 7.07)	-
Tribe	0.03 (0.001 to 0.05, 0.05, 7.20)	0.03 (-0.02 to 0.08, 0.27)
Pelvis height	1	-

+ Constant = 7.97 (6.37 to 9.56, <0.001)

On multilevel multivariable regression modeling of all the variables controlling for site of birth, maternal age remained non-significant and was dropped due to its additional strong correlation with number of pregnancies (gravidity). Maternal tribe though non-significant was retained in the final model as a proxy of maternal genotype. Table 4 shows the other variables in the final model in which there was a 0.01Cm reduction in maternal pelvis height for each centimeter increase in maternal height (P=0.02), and a 0.01 centimeter increase in maternal pelvis height for each kilogram increase in maternal weight (P<0.01). Finally there was a 0.04-centimeter increase in maternal pelvis height for each additional pregnancy (P=0.03). The ICC for a 2 level model, “a”, with the above measurements with respect to tribe was 0.20 (SE=0.08, 95% CI 0.09 to 0.38); while for a two level model, “b”, with respect to site was 0.37 (SE=0.11, 95% CI 0.18 to 0.60). A three level model, “c”, had an ICC of 0.37 (SE=0.11, 95% CI 0.18 to 0.60) for tribe of mother nested in site of birth while the higher-level site of birth had an ICC of 0.36 (SE=0.11, 95% CI 0.18 to 0.60). Comparisons of the log likelihood ratios for models “c” and “b” gave a chi square value of 1.33 (p=0.27).

## Discussion

We set out to determine the associations between maternal: Age, height, weight and number of pregnancies with maternal pelvis height in Ugandan mothers. We found significant associations between maternal pelvis height and maternal height, weight and number of pregnancies (see Table 4).

The significance of the observed reduction in maternal pelvis height in relation to increasing maternal height is especially important in view of earlier work described elsewhere, that demonstrated that a greater maternal height is associated with better pregnancy outcomes [5, 8]. Where the better maternal height (taller mothers) is associated with both bigger genotypic size and better feeding during childhood on the part of the mother, results in bigger dimensions of the maternal birth canal that in turn should lead to easy passage of a normal sized fetus for this mother [5, 7, 8]. From this study we observe that the inverse relationship between maternal height with maternal pelvis height may impact the process of childbirth through its influence on the inclination of the pelvis [11]. Since pelvis height increases proportionally with pelvis incidence these taller mothers will have less inclined birth canal inlet. This less inclined birth canal inlet is in turn more receptive for the descending fetal head. Thus in the presence of an adequately sized birth canal, as is expected within a taller mother, we find explanations for the previously described: increased likelihood of fetal head engagement during the antenatal period [9], reduced chances of fetal head engagement [10] and hypothesized shorter duration of labor. On the other hand, a short woman with a larger pelvis height is also at an advantage since the larger pelvis height is also associated with larger birth canal dimensions [11]. The above advantages improve with increasing number of pregnancies and thus age since the pubis bone in female continues growing till the 30th year of life compared with other bones that stop by the 20th year for the rest of the body [15]. At this point it is important to note that being tall (greater maternal height) or having a large pelvis height both lead to better childbirth outcomes.

The challenge thus remains for the short woman with a small pelvis height with respect to possible increased occurrence of adverse outcomes of pregnancy. In this study we observed a degree of clustering of the various measurements (see Figure 2 map). At one of the sites this combination of short mothers with small maternal pelvis heights was very common (see also Table 2). For such a site one would expect, and indeed we observed, an increased risk of fetal head molding and adverse outcomes of childbirth [10]. Whereas this risk may be low for the individual mother [8], at the population level such observations are of public health interest more so in relation to the distribution of health services to mitigate this risk. This public health perspective may be even stronger if these measurements were linked to the early childhood feeding of the mother. This linkage must factor in the effect of migration, since migration has been associated with worse childbirth outcomes as has been seen with Ethiopian Jews [16], and Africans giving birth in foreign countries [17]. In these developed settings the improved feeding during pregnancy may work with the unborn fetal genetics to give cephalo-pelvic disproportion as a result of child that is big or in some cases normal sized but large for the mothers’ birth canal. In this study we did not assess for local migration as such were unable to extrapolate the study finding as suggested above. This limitation does not dampen the argument for the potential public health application of these measurements in view of the higher and more stable correlation (ICC) for measurements with respect to site of childbirth (see also Figure 2). The correlations due to site of childbirth were stronger than those seen for tribe of mother, a measure of ethnicity and thus a proxy for the maternal genotype. This of course assumes minimal migration for this multiethnic population.

Some of the other limitations of this study include the challenge related to obtaining the anthropometric measurement whose accuracy may not be as high due to possible variability in the caliber and experience of people that run these units. To minimize this potential source of bias the team ensured that all the nurses participating in the study were experienced with attending to mothers and received both the initial additional refresher training in how to measure the maternal pelvis height. The study findings are also based on measurements made on mothers who come to use the health facilities [3]. This may further reduce the generalizability of the study findings as such there is need for caution with regards to extrapolating these study findings for application to the general population.

## Conclusion

In conclusion this study shows that maternal pelvis height was associated with maternal height, maternal weight and number of pregnancies. The study measurements were in turn affected by the site of childbirth followed by the tribe of the mother. There is need for more study to further identify the site-specific factors affecting these measurements so as to increase the public health value of such assessments in these low resource settings.

### What is known about this topic

- Sub-Saharan Africa remains challenged by the very high numbers of birth associated maternal and child deaths;
- Most mothers in this region will come to the health facility for antenatal care at least once during pregnancy, which makes this visit a valuable screen opportunity;
- Maternal pelvis height is an anthropometric measurement, already being used by automotive engineers, that has be shown to have significant associations with various maternal and fetal birth related events/outcomes.

### What this study adds

- This study demonstrates the significant relationships between maternal pelvis height and other anthropometric measurements in a large multisite and multiethnic population;
- The study also highlights the public health value of these measurements when used in combination to aid the screening for adverse events of parturition at the population level.
